# Effect of acupuncture on BDNF signaling pathways in several nervous system diseases

**DOI:** 10.3389/fneur.2023.1248348

**Published:** 2023-09-14

**Authors:** Chenxin Miao, Xiaoning Li, Yishu Zhang

**Affiliations:** ^1^Second Clinical Medical School, Heilongjiang University of Chinese Medicine, Harbin, Heilongjiang, China; ^2^Department of Acupuncture, The Second Affiliated Hospital of Heilongjiang University of Chinese Medicine, Harbin, Heilongjiang, China

**Keywords:** acupuncture, BDNF, nervous system diseases, signaling pathways, mechanism

## Abstract

In recent years, the understanding of the mechanisms of acupuncture in the treatment of neurological disorders has deepened, and considerable progress has been made in basic and clinical research on acupuncture, but the relationship between acupuncture treatment mechanisms and brain-derived neurotrophic factor (BDNF) has not yet been elucidated. A wealth of evidence has shown that acupuncture exhibits a dual regulatory function of activating or inhibiting different BDNF pathways. This review focuses on recent research advances on the effect of acupuncture on BDNF and downstream signaling pathways in several neurological disorders. Firstly, the signaling pathways of BDNF and its function in regulating plasticity are outlined. Furthermore, this review discusses explicitly the regulation of BDNF by acupuncture in several nervous system diseases, including neuropathic pain, Parkinson’s disease, cerebral ischemia, depression, spinal cord injury, and other diseases. The underlying mechanisms of BDNF regulation by acupuncture are also discussed. This review aims to improve the theoretical system of the mechanism of acupuncture action through further elucidation of the mechanism of acupuncture modulation of BDNF in the treatment of neurological diseases and to provide evidence to support the wide application of acupuncture in clinical practice.

## 1. Introduction

As one of the main constituents of traditional Chinese medicine, acupuncture has been used to treat various diseases in China and other Asian countries for more than 3,000 years (1). In 2010, The United Nations Educational, Scientific, and Cultural Organization added acupuncture to the Representative List of the Intangible Cultural Heritage of Humanity (2). Acupuncture is the discipline of studying meridians, acupoints, and acupuncture therapy (3). The theoretical basis of acupuncture is the meridian system. Meridian is the channel for the flow of vital energy, or “qi,” throughout the body (4). When the flow of qi is interrupted, disease ensues (5). Acupuncture works by stimulating specific areas of the body with acupuncture needles to regulate the flow of qi and blood and to rebalance the flow of energy and blood (6). During acupuncture, needle rotations cause increased tension and changes in connective tissue structure. These changes lead to various cellular and extracellular events (6). A groundbreaking development in acupuncture was the development of electroacupuncture (EA). EA is a combination of needles inserted into the skin or underlying muscles followed by electrical stimulation, which can achieve similar or better clinical efficacy than manual needling (7). EA can activate neuronal networks, thus modulating the function of certain organs to treat various diseases (8).

According to numerous studies, acupuncture has a variety of physiological effects, including analgesic, muscle-relaxing, anti-inflammatory, mildly anxiolytic, and antidepressant effects. These biological effects are mediated by biological processes, including central sensitization, neurotransmitters, intestinal flora, immunomodulation, oxidative stress, and neuroinflammation (1). Although there is an emerging understanding of the pathophysiology of acupuncture in treating neurological disorders, the mechanisms of acupuncture treatment and the BDNF signaling pathways have not been elucidated.

This review will discuss the modulation of BDNF by acupuncture in several neurological diseases. For instance, EA improves neuropathic pain by blocking BDNF/TrkB (tropomyosin-related kinase B) signaling pathway-mediated central sensitization (9), and acupuncture activates PI3K (phosphoinositide 3-kinases) /Akt (protein kinase B) and MEK (mitogen activated protein kinase kinase) /ERK (extracellular signal–regulated kinases) signaling pathways downstream of BDNF, thereby promoting the survival of dopaminergic neurons and improving the symptoms of Parkinson’s disease (10). Microglia activated after cerebral ischemia secrete more BDNF, which binds to its high-affinity receptor TrkB to protect during the acute stroke and stimulate neural repair at a later stage (11, 12). In addition, homeostatic plasticity of BDNF in the hippocampus may be related to the mechanism of acupuncture in the treatment of depression (13).

## 2. The genesis of BDNF

BDNF is the most extensively studied neurotrophic factor in the mammalian nervous system. It plays an essential role in signal transduction, neuronal development, central nervous system plasticity regulation, and treatment of neurological diseases (14–16). Barde et al. (17) first documented the existence of a component that promotes the survival of embryonic sensory neurons in the glioma-conditioned media in 1978. Subsequently, in 1982, the team purified the factor from the porcine brain and named it BDNF. As a result, following NGF (nerve growth factor), BDNF was the second neurotrophic factor to be isolated (17). In the following years, other neurotrophic factors were discovered successively, and the neurotrophic family now includes NGF, BDNF, neurotrophic factor 3 and neurotrophic factor 4, and neurotrophic factor 5 (18–21).

## 3. BDNF synthesis, signaling pathway, and function

Since its discovery, BDNF has been studied spanning 40 years. In recent years, the focus of attention on BDNF has been on molecular biology and signaling pathways.

### 3.1. BDNF synthesis and receptors

In molecular biology, the earliest attention was focused on the rodent BDNF gene, which, although similar to the human BDNF gene, also has many noticeable structural differences. It was not until 2007 that several key features of the human BDNF gene were discovered (22). The BDNF gene consists of 11 exons, each of which maintains a specific translation pattern to alter the length of the translated BDNF peptide, and the expression of different BDNF peptides causes different biological effects. There are at least three functional BDNF peptides, namely pro-BDNF (proneurotrophin isoform of BDNF), m-BDNF (mature brain-derived neurotrophic factor), and pro-domain (23).

**Figure 1:**
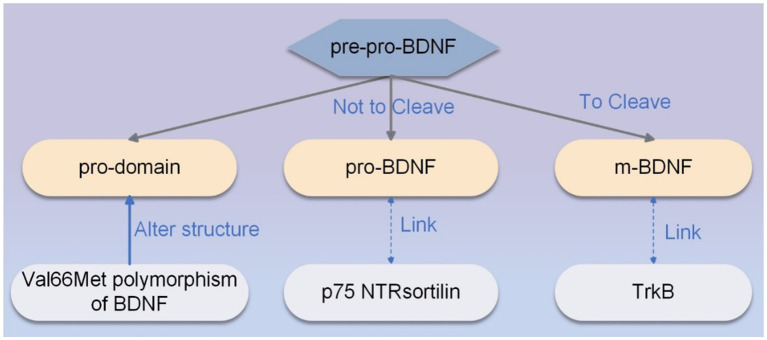
The synthesis pathway of BDNF. The primary sequence of pre-pro-BDNF is divided into functionally active isoforms of pro-domain, pro-BDNF, and m-BDNF, each of which exhibits specific affinity for a particular type of receptor. M-BDNF binds with high affinity to the TrkB receptor, and pro-BDNF is secreted as a functional protein that binds to the p75 NTR-sortilin receptor complex. The Val66Met polymorphism of BDNF can alter the structure of the pro-domain, modulate neuronal morphology, and induce the retraction of neuronal growth cones.

BDNF is synthesized as pre-pro-BDNF, which is processed in the secretory pathway or can be cleaved by plasma proteins or metalloproteinases and secreted to generate m-BDNF. M-BDNF binds affinity to the TrkB receptor highly (24). If pre-pro-BDNF is not cleaved within the endoplasmic reticulum or vesicles (25), pro-BDNF, the heterodimer of pre-pro-BDNF, is secreted as a functional protein that binds to the p75 NTR (p75 neurotrophin receptor)- sortilin receptor complex (23). Pro-BDNF is considered to be important in regulating brain function during brain development, whereas m-BDNF affects neuroprotection and synaptic plasticity in adulthood (26). Notably, the ratio of pro-BDNF to m-BDNF is affected at different times and regions of brain development, and this ratio in excitatory and inhibitory neurons can be used as a biomarker of neuropsychiatric and neuropathological conditions (27). The Val66Met polymorphism of BDNF can alter the structure of pro-domain, regulate neuronal morphology, and induce the retraction of neuronal growth cones (28). In addition, the Val66Met polymorphism of BDNF also affects activity-dependent secretion of BDNF and memory and hippocampal function in humans (29). The synthesis pathway of BDNF is shown in Figure 1.

### 3.2. BDNF signaling pathways and functions

In terms of signaling pathways, different BDNF isoforms with distinct types of receptors trigger functionally diverse signaling pathways. Activating these signaling pathways is crucial for sustaining the dynamic equilibrium between stimulatory and inhibitory effects throughout cerebral maturation, synaptic plasticity, and post-injury brain restoration.

**Figure 2:**
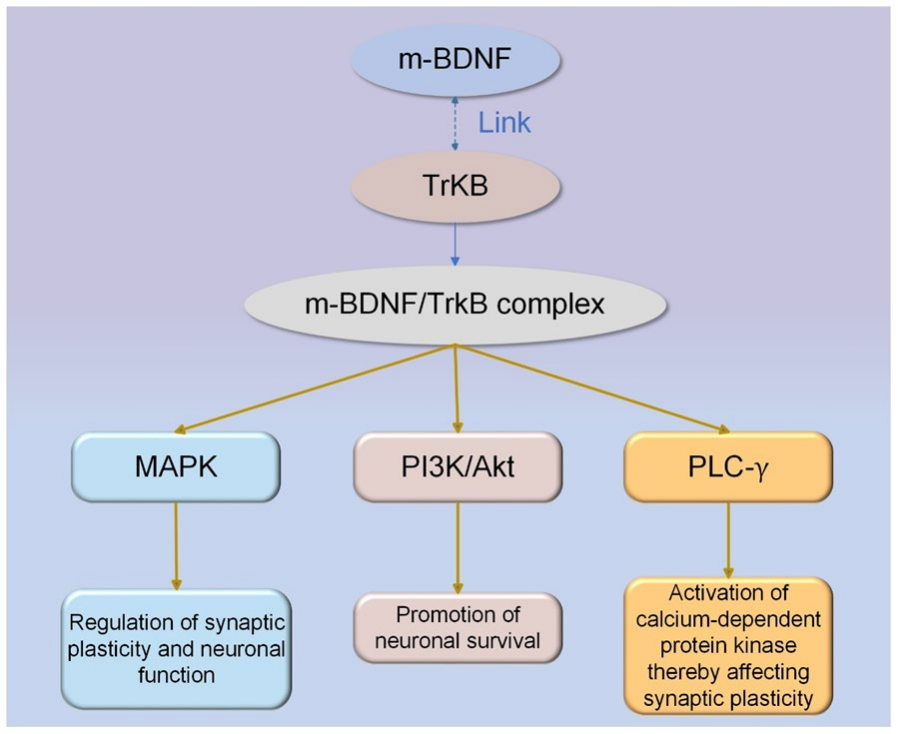
The BDNF/TrkB signaling pathway. M-BDNF isoforms interact with TrkB receptors to activate intracellular signaling cascades. M-BDNF/TrkB receptor complexes trigger MAPK, PI3K/Akt, and PLC-γ signaling pathways, and each pathway has its specific function.

Firstly, activation of TrkB by BDNF initiates numerous downstream pathways essential for neuronal survival and function. TrkB activation occurs through trans-phosphorylation of its tyrosine residues, leading to activation of src homologous domain 2 and initiation of signaling through MAPK (mitogen-activated protein kinase), PI3K/Akt, and PLC-γ (phospholipase C-γ) pathways (30–32). The MAPK signaling pathway facilitates synaptic plasticity and neuronal function regulation, whereas PI3K/Akt activation enhances neuronal survival. Additionally, the PLC-γ pathway enables calcium-dependent protein kinases to be activated, which has implications for synaptic plasticity (33). The BDNF/TrkB signaling pathway is shown in Figure 2.

**Figure 3:**
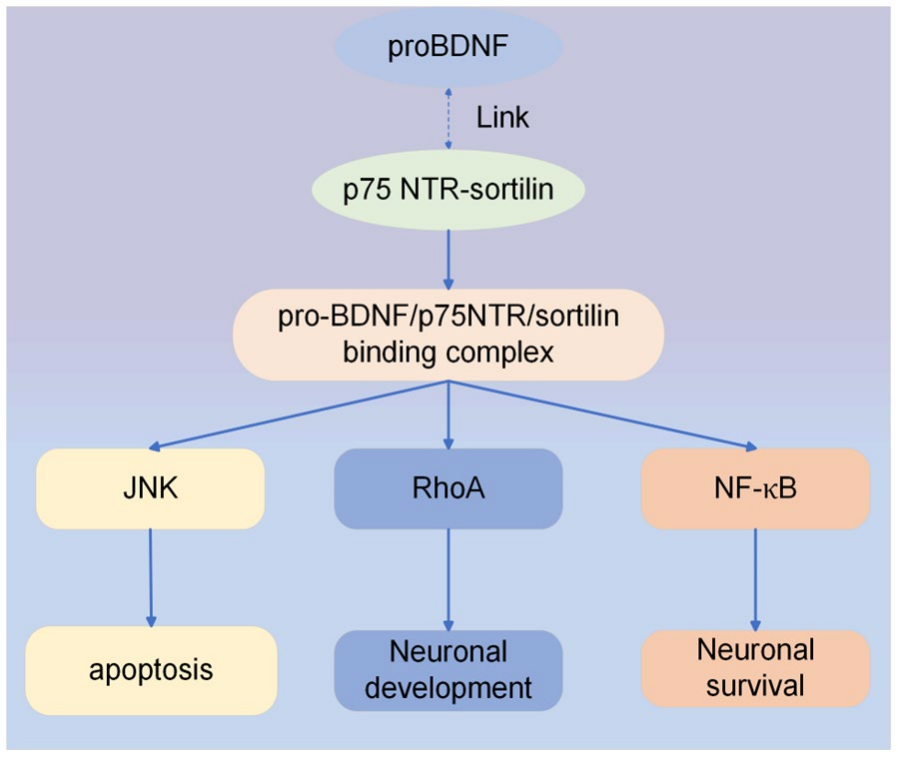
ProBDNF/p75NTR signaling pathway. Intracellular signaling cascade activated by pro-BDNF heterodimer interaction with p75NTR and sortilin receptors. Formation of the proBDNF/p75NTR/sortilin binding complex triggers signaling pathways associated with JNK, RhoA, and NF-κB.

Secondly, pro-BDNF binds to the p75 NTR-sortilin complex when not cleaved. Pro-BDNF/p75NTR/sortilin binding complex initiates a signaling cascade leading to the activation of c-Jun amino-terminal kinase (JNK), Ras homologous gene family member A (RhoA), and NF-κB (nuclear factor kappa B) (28, 34). JNK-related pathway can induce apoptosis (35), the RhoA signaling pathway regulates neuronal development (34), and the NF-κB pathway can promote neuronal survival (34). Recently, research has shown that the activation of the pro-BDNF/p75NTR signaling pathway in the spinal cord contributes to the alleviation of inflammatory pain (36). In addition, a clinical trial found that genetic deficiency through p75NTR and thus blocking upregulated pro-BDNF inhibited the differentiation of antibody-secreting cells, which ultimately reduced the disease activity in systemic lupus erythematosus (37). The effect of the pro-BDNF/p75NTR signaling pathway has been less studied, but its therapeutic effects on the nervous system cannot be ignored. Pro-BDNF/p75NTR signaling pathway is shown in Figure 3.

In addition, neuroinflammation also affects BDNF-related signaling pathways, causing psychiatric and neurodegenerative disorders to exhibit abnormal BDNF levels (38). Because of this, modulating BDNF levels could be a potential therapeutic tool for neurological disorders (39).

## 4. BDNF and plasticity

Synaptic plasticity is a property of synapses that is regulated in such a way that synaptic form and function are strengthened or weakened in response to the strengthening or weakening of their own activity. It has been shown to be the biological basis for cellular learning memory activity (40). Many researches have confirmed the essential role of BDNF in regulating synaptic plasticity (26, 41). Specifically, BDNF regulates synapse formation in three essential manners: increasing axon and dendrite branching, inducing axon and dendrite formation, and stabilizing existing synapses (42). Recent studies have found that BDNF affects synapse formation temporally and spatially (42).

From a temporal perspective, Hebbian plasticity is the most extensively researched form of activity-dependent adaptation to synaptic strength, encompassing long-term potentiation and depression (LTP and LTD) and presynaptic potentiation and depression. LTP and LTD are the fundamental models of learning memory activity at the cellular level and synapse-related mechanisms that regulate functional recovery after neural injury. LTP is a presynaptic neuron that induces a prolonged increase in the amplitude of postsynaptic potentials after a short period of rapid high-frequency repetitive stimulation. LTD is a postsynaptic neuron that produces a prolonged decrease in the amplitude of postsynaptic potentials after sustained low-frequency stimulation of the anterior neuron (43). Many studies have demonstrated the mediation of LTP, synaptic plasticity, and learning in the developing and adult hippocampus by BDNF (44, 45). Lin et al. found that BDNF/TrkB signaling can regulate LTP in the hippocampus. Prograde BDNF/TrkB signaling could potentially contribute to the generation of LTP, and prograde and retrograde BDNF/TrkB signaling could then persist both pre-synaptically and post-synaptically to maintain LTP. Thus, LTP is induced by presynaptic BDNF and postsynaptic TrkB, while postsynaptic BDNF and presynaptic TrkB maintain LTP (46). Interestingly, m-BDNF binding to TrkB promotes LTP while inhibiting LTD. In contrast, pro-BDNF facilitates N-methyl-D-aspartic acid receptor-dependent LTD in the hippocampus through activation of the p75NTR, suggesting a bidirectional regulation of synaptic plasticity by pro-BDNF and m-BDNF (47).

From a spatial viewpoint, BDNF is an essential mediator of homeostatic plasticity (48, 49). Homeostatic plasticity refers to the maintenance of activity near a set point that serves to stabilize neurons or neuronal circuits in the presence of perturbations that alter excitability, such as changes in cell size, synaptic number, or strength (49). Perturbations of excitability are known to modulate BDNF expression. BDNF affects the phenotype of GABAergic interneurons that release inhibitory neurotransmitters, which in turn modulates the excitability of cortical circuits and ultimately regulates cortical inhibition through BDNF-dependent mechanisms. It is suggested that BDNF plays vital in controlling cortical excitability and stabilizing neuronal activity (50).

Notably, the synaptic substrate for learning and memory is provided by Hebbian plasticity, whereas network activity is stabilized by homeostatic plasticity. These two forms of plasticity interact functionally and both contribute to the regulation of plasticity by BDNF (51). Therefore, it is vital to consider the temporal scale and spatial location for BDNF to regulate synaptic plasticity (42).

As mentioned earlier, activation of TrkB by BDNF triggers the MAPK and PLC-γ pathways, which are relevant to synaptic plasticity. Activation of MAPK signaling regulates neurotransmitter release, and PLC-γ signaling pathway activates calcium-dependent protein kinases, thereby affecting synaptic plasticity. Calcium levels play an important role in neurotransmission. A wealth of evidence has shown that BDNF can induce elevated calcium levels at the cellular and synaptic levels (52, 53). Notably, the effect of BDNF/TrkB signaling on calcium signaling varies with the location of brain regions (42).

Furthermore, it has also been found that there is a crosstalk between 5-HT (5-hydroxytryptamine) as a regulator of neuroplasticity and BDNF, which can control the function of each other through common intracellular signaling pathways. The balance of the 5-HT/BDNF system may play a vital role in the emergence of both healthy and diseased phenotypes. Conversely, if the balance is disrupted, it can lead to depression, suicide, and other behaviors (54).

Despite numerous studies discussing BDNF, there are fewer studies on BDNF in treating diseases with acupuncture. The effects of acupuncture on BDNF in various neurological diseases will be discussed separately below.

## 5. Effects of acupuncture on BDNF in different neurological disorders

**Figure 4:**
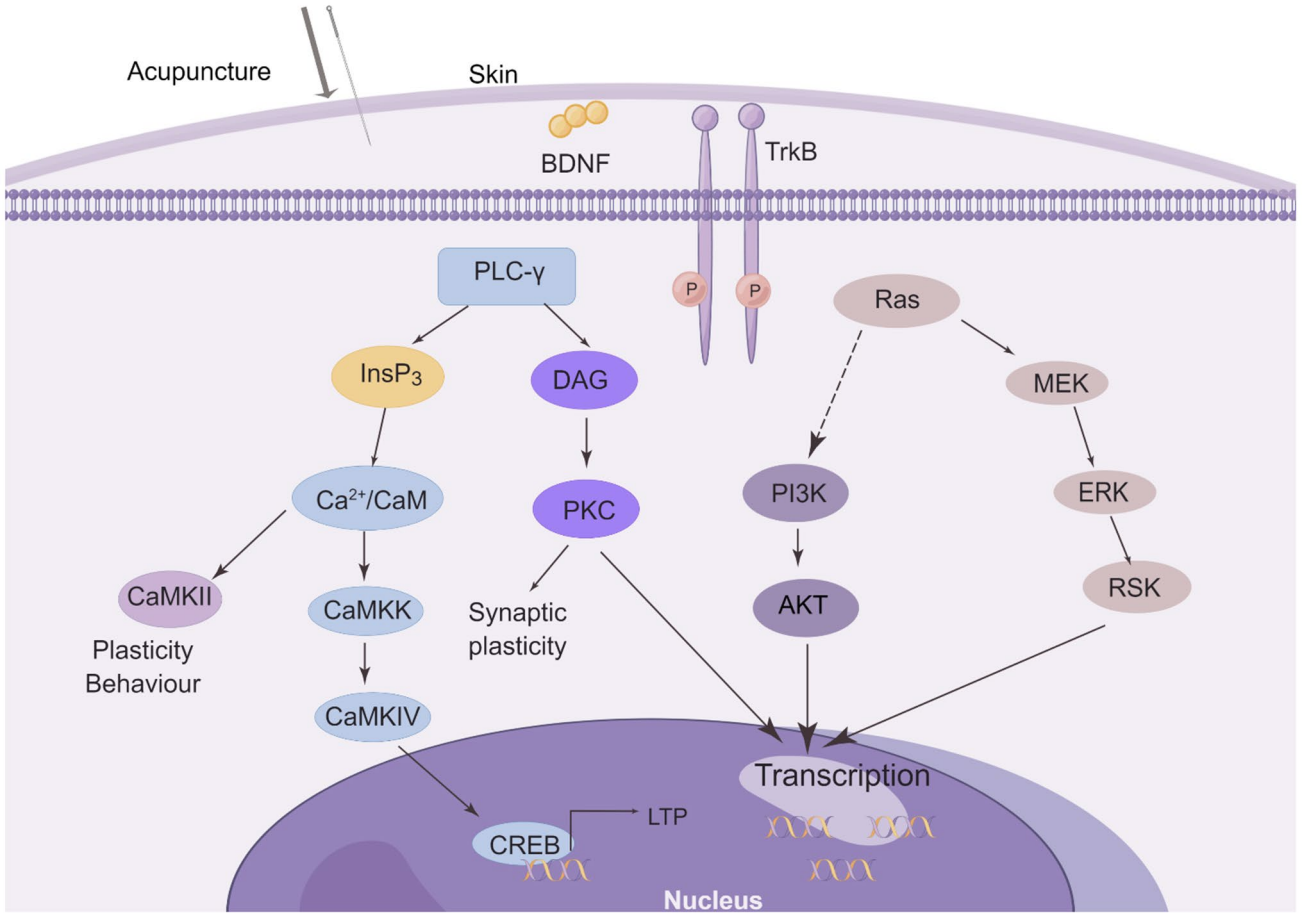
The effect of acupuncture on the BDNF/TrkB signaling pathway. Three major intracellular signaling pathways are activated after needling. The first pathway is the activation of the Ras-mitogen-activated protein kinase (MAPK) signaling cascade, which promotes neuronal differentiation and growth through MAPK/ERK kinase (MEK) and extracellular signal-regulated kinase (ERK). It activates the phosphatidylinositol 3-kinase (PI3K) cascade. Another pathway is the activation of phospholipase C-γ1 (PLC-γ1), which leads to the production of inositol trisphosphate (InsP3) and diacylglycerol (DAG). Whereas DAG stimulates protein kinase C (PKC) isoforms, InsP3 promotes Ca2+ release from internal stores and subsequent activation of Ca2+/calmodulin (Ca2+/CaM)-dependent protein kinases (CaMKII, CaMKK, and CaMKIV) These signaling pathways all regulate gene transcription and some may be involved in LTP. (This figure is drawn by Figdraw).

Acupuncture demonstrates positive therapeutic effects on numerous neurological disorders by activating BDNF and its downstream signaling pathways. The effect of acupuncture on the BDNF signaling pathway is shown in Figure 4.

### 5.1. Neuropathic pain

Neuropathic pain is one of the most challenging medical problems. It is caused by lesions and diseases of the somatosensory system (55). The primary somatosensory cortex (S1) is essential for sensory processing, and neuropathic pain is linked to its maladaptive plasticity. BDNF from cortical microglia promotes neuroplasticity in the somatosensory cortex and enhances hypersensitivity to neuropathic pain (56). In addition, there is increasing evidence that BDNF may act not only as a pro-nociceptive factor but also as an antinociceptive neurotrophic factor (24). In higher brain areas, neuropathic pain may be relieved by EA via altering excitatory and inhibitory neurotransmitters (57).

Central sensitization is linked to neuropathic pain. By mediating LTP in the spinal cord and controlling synaptic plasticity, BDNF contributes to central sensitization in the spinal cord (58). In the spinal cord of rats with sparing nerve injury (SNI), 2 Hz EA dramatically decreased BDNF and TrkB mRNA and protein expression levels. Additionally, neuropathic pain was reduced by inhibiting the central sensitization caused by the BDNF/TrkB signaling pathway (9).

In 2018, Tu et al. (59) used EA to stimulate Yang Lingquan acupoint (GB-34) and Zu Sanli acupoint (ST-36) in the posterior spinal cord of chronic constriction injury (CCI) rats, which is the most commonly used animal model for neuropathic pain. The findings indicated that EA significantly reduced the ratio of activated microglia and the mRNA and protein expression levels of BDNF and TrkB in the posterior spinal cord of CCI mice. EA was thought to reduce neuropathic pain by preventing microglia in the spinal cord from becoming activated and subsequently blocking the BDNF/TrkB signaling pathway. Hereafter, their group focused on the miRNA-BDNF network to explore the effects of EA on this network. Previous studies have shown that miRNAs are associated with regulating pain-related networks. In this study, miR-206-3p was shown to be negatively regulated by EA therapy, which decreased the expression of the target gene BDNF while upregulating miR-206-3p. It ultimately alleviated CCI-induced neuropathic pain (60).

In male hyperalgesic priming (HP) model rats, EA dramatically raised the mechanical pain threshold, decreased the overexpression of BDNF and p-TrkB, increased the expression of K + -Cl-Cotransporter-2, and reduced abnormal mechanical pain (61). It implies that EA can interfere in pain conversion and be a helpful treatment for preventing the change from acute to chronic pain.

According to the above results, the mRNA and protein expression levels of BDNF and TrkB are considerably downregulated by EA, which reduces neuropathic pain.

### 5.2. Parkinson’s disease

The pathology of Parkinson’s disease is characterized by a progressive loss of dopaminergic neurons in the substantia nigra of the brain, culminating in a severe deficiency of striatal dopamine and the major motor symptoms of Parkinson’s disease (e.g., resting tremor and bradykinesia). BDNF is a potential drug for treating Parkinson’s disease since it has been shown to have neuroprotective and neurorestorative effects on dopaminergic neurons. As demonstrated in animal models, it can somewhat protect neurons from neurotoxic attacks (62).

Zhao et al. (10) found that acupuncture can inhibit apoptotic pathways, upregulate neuro factor expression, and activate downstream PI3K/Akt and MEK/ERK pathways, thereby promoting the survival of dopaminergic neurons. Through antioxidant, anti-inflammatory, and anti-apoptotic mechanisms, acupuncture can protect dopaminergic neurons from deterioration and control neurotransmitter homeostasis in basal ganglia circuits.

MPTP(1-methyl-4-phenyl-1,2,3,6-tetrahydropyridine)-induced Parkinson’s disease in mice was treated with EA at GB34 (Yang Lingquan) and LR3 (Tai chong) acupoints. Through the activation of the survival pathway of Akt and BDNF in the substantia nigra area and an increase in striatal dopamine levels, EA reduced MPTP-induced rotational behavior and motor activity (63).

In subsequent experiments (64), MPTP-induced mice were still selected as the animal model, but the acupoints were changed to GV20 (Bai hui); GV29 (Yin Tang) treatment, and the results showed that EA regulated and activated downstream pathways (such as PI3K/ Akt and ERK1/2) via the TKB FL/TrkB T1 ratio to counteract MPTP-induced Parkinson’s disease in mice, and upregulated BDNF expression against the neurotoxicity of MPTP. EA improves motor dysfunction and reduces the degeneration of dopaminergic neurons.

EA treatment at GB34 (Yang Lingquan) and BL60 (Kunlun) prevented MPTP-induced reduction in striatal BDNF and phosphorylated-ERK expression. It indicates that EA prevents nigrostriatal neuronal death and restores neurogenesis in the SVZ (subventricular zone). EA can potentially promote SVZ neurogenesis by activating BDNF/ERK signaling (65).

The results of the animal experiments described above show that the same choice of EA treatment, even with different options of acupuncture points and treatment parameters, results in an upregulation of BDNF expression in the end, improving motor symptoms and dopaminergic neurodegeneration in Parkinson’s disease.

### 5.3. Cerebral ischemia

The acute characteristic of ischemic stroke is the massive and rapid loss of axons (66). While acupuncture increases synaptic structure and function, axonal germination and regeneration, and neurogenesis. These processes modulate the neural network and some of the damaged areas of the brain, increasing various abilities and adaptations (67).

BDNF has emerged as a critical regulator of neuroplasticity in motor learning and healing after stroke, although many molecular signaling pathways are involved in recovery after stroke (68). Microglia activated after cerebral ischemia secrete more BDNF, which exerts its effects through binding to its high-affinity receptor TrkB (11). Notably, BDNF/TrkB would play a protective role in acute stroke and stimulate nerve repair at a later stage (12). It has been shown that acupuncture can upregulate the expression of endogenous BDNF and stem cell factors in areas such as the cerebral cortex, hippocampus, and caudate cone after cerebral ischemia, which facilitates the repair of neuronal damage (69).

EA treatment at Bai hui (GV20), Da zhui (GV14) starting 1 day after cerebral ischemia–reperfusion effectively increased BDNF expression, provided BDNF-mediated neuroprotection and prevented caspase-3-dependent neuronal apoptosis through phosphorylation of Raf-1/MEK1/2/ERK1/2/p90RSK/Bad signaling cascade (70). Following ischemic stroke therapy with EA, increased neural stem cell proliferation and differentiation via BDNF and VEGF signaling pathways encourage functional recovery (71).

Acupuncture at Bai hui (GV20) and Si shencong (Ex-HN1) significantly enhanced growth and development, improved neurobehavioral functions, learning and memory abilities. It also attenuated apoptosis and upregulated levels of GDNF (glial cell line-derived neurotrophic factor) and BDNF in the hippocampus (72). Inhibiting apoptosis and boosting GDNF and BDNF expression levels in the hippocampus of hypoxia-ischemia -exposed rats are two ways that acupuncture may be used as a treatment.

In a rat ischemia–reperfusion injury model, EA reversed the reduction of BDNF induced by ischemia–reperfusion injury and enhanced the BDNF/TrkB pathway to promote neuroprotection (73).

By increasing cerebral blood flow to ischemic regions and encouraging the growth of CNS (central nervous system) cells, acupuncture preserves CNS neurons and promotes functional remodeling of the CNS. Acupuncture exerts a dual effect of alleviating injury and promoting growth by regulating BDNF (74).

### 5.4. Depression

Depression is managed by two systems in the brain. Firstly, the brain’s stress system, the hippocampus-HPA (hypothalamus-pituitary adrenocortical) pathway. The functional parts of learning and memory, as well as the inhibition of HPA-mediated stress pathways, are regulated by the hippocampal circuitry, both of which are disrupted in depression. Secondly, the behavior of depressed patients is controlled by the brain reward system, that is, the VTA (ventral tegmental area)- NAc (nucleus accumbens) and VTA-prefrontal cortex pathways, and the dopaminergic VTA-NAc pathway is essential for motivation and reward. Stress can lower BDNF levels in the brain, resulting in hippocampal and prefrontal cortex atrophy, cell death, and severe depression (75). There is emerging evidence that various intracellular pathways and signaling cascades are associated with the pathophysiology and treatment of depression (76). Homeostatic plasticity of BDNF in the hippocampus may be involved in the therapeutic mechanisms of psychiatric disorders (13).

In many clinical studies, EA has shown powerful antidepressant effects (77). To treat depression, EA can inhibit hyperexcitability of the HPA axis, decrease levels of inflammatory cytokines, enhance BDNF expression, restore hippocampal synaptic plasticity, regulate neuropeptides and neurotransmitters bidirectionally, and stimulate signal transduction pathways and genomic expression (78).

Chronic stress as a cause of onset, maintenance or exacerbation of depression. A significant increase in tissue fibrinogen activator (tPA), BDNF and BDNF mRNA concentrations in the hippocampus was observed in chronic unpredictable mild stress (CUMS) rats treated with EA (79). This indicates that EA prompts antidepressant effects by activating the tPA/BDNF pathway within the hippocampus.

In addition, EA at Yin tang (EX-HN3) and Bai hui (DU20) increased the phosphorylation of cyclic adenosine phosphate response element binding protein and the level of BDNF, which promoted neural regeneration and exerted antidepressant effects (62). EA at Bai hui (GV20), Nei guan (PC6) and San yinjiao (SP6) significantly increased BDNF expression in the serum and hippocampus. EA can produce antidepressant effects by promoting BDNF expression and improved behavioral responses (80).

One study indicated that major depressive disorder patients had considerably reduced levels of BDNF pro-domain. Pro-domain of BDNF promotes LTD in the hippocampus and regulates the mechanisms required to promote synaptic inhibition (81). EA increases CREB phosphorylation and BDNF expression by regulating the CREB pathway (TrkB-CREB-BDNF, PKA-CREB-BDNF, and CaMKII-CREB-BDNF) thus causing antidepressant effects (82). BDNF is involved in the plasticity of numerous neurons throughout various brain regions and significantly impacts stress-related psychiatric disorders (83). Acupuncture appears to have a significant effect on the emergence of depression by boosting the production of the plasticity protein BDNF.

### 5.5. Spinal cord injury

Previous studies have found that EA treatment of spinal cord injury can significantly increase the expression of BDNF and TrkB proteins and downregulate the expression of p75NTR proteins, but the intrinsic connection is not clear. Further investigation revealed that EA can make BDNF bind to TrkB with high affinity and promote the repair of spinal cord injury. At the same time, the proBDNF-p75NTR signaling pathway was inhibited to achieve nerve regeneration and repair (84). Mature neurotrophic proteins bound to BDNF and p75NTR could activate the JNK pathway, triggering cell death through the activation of p53 (as shown in Figure 3).

Tu et al. (85) found that EA could effectively elevate the expression level of BDNF and promote the repair of damaged nerves after treating the spinal cord injury model rats with EA on Da zhui (GV 14) and Ming men (GV 4).

### 5.6. Others

EA Jingming (BL1) and Shuigou (GV26) may inhibit retinal ischemia–reperfusion injury Retinal ischemia–reperfusion injury RIRI-induced inflammation by activating the DOR-BDNF/TrkB pathway to protect vision (86).

In the rat model of insomnia, EA was used to stimulate Baihui (DU20), Shenmen (HT7), and San yinjiao (SP6), EA stimulates acupoints of sleep factors, cAMP/CREB/BDNF, PI3K/Akt pathways and the multipath and multitarget body response regulation mechanism of apoptosis (87).

The effect of acupuncture on BDNF and downstream signaling pathways in certain neurological disorders are shown in Table 1.

**Table 1:** Effect of acupuncture on BDNF signaling pathway in different nervous systems.

Model	Intervention	Acupoints	Acupuncture parameter	Signal pathway	Result	References
Neuropathic pain in SNI rats	EA	/	2 Hz	BDNF/TrκB signaling	Down-regulated the expression levels of BDNF mRNA and protein	(9)
CCIinduced neuropathic pain rats	EA	GB34/ST36	2/100 Hz, 1.5 mA, 30 min	BDNF–TrkB pathway	Decreased the expression of BDNF and TrkB at both the mRNA and protein levels	(59)
CCIinduced neuropathic pain rats	EA	GB34/ST36	2/100 Hz, 1.5 mA, 30 min, every 24 h from the 8th day after CCI, for a total of 7 days.	/	The expression levels of BDNF decreased The expression level of miR-206-3p elevated	(60)
HP model	EA	ST36/BL60	2/100 Hz, 0.5 mA, 1.0 mA and 1.5 mA intensity (intensity increased every 10 min) for a total of 30 min	BDNF–TrkB pathway	Inhibit BDNF expression and TrkB phosphorylation	(61)
MPTP-lesioned Parkinsonism mice	EA	GB34/LR3	0/50 Hz,1 mA,20 min	/	Increase the expression of BDNF	(63)
MPTP-lesioned Parkinsonism mice	EA	GV20/GV29	2 Hz, 1.5 mA, 10 min	BDNF/TrkB signaling pathway	Up-regulated BDNF expression	(64)
MPTP-lesioned Parkinsonism mice	EA	GB34/BL60	2 Hz/1.5 mA/20 min for 3 weeks	BDNF-ERK signaling	Activation of BDNF/ERK signaling	(65)
MCAO rats	EA	GV20/GV14	5 Hz, 2.7 mA-3.0 mA, 25 min once daily	BDNF-mediated MEK1/2/ERK1/2/ p90RSK/bad signaling pathway	Up-regulated BDNF expression	(70)
MCAO mice	EA	GV20/GV14	2 Hz, 2 volts, 20 min	BDNF and VEGF signaling pathway	BDNF significantly increased	(71)
Hypoxia–ischemia in neonatal rats	Manual acupuncture	GV20/Ex-HN1	Two spins, second for 15 s retained for 30 min	/	Up-regulated BDNF and GDNF expression in hippocampus	(72)
CUMS rats	EA	DU20/EX-HN3	2 Hz, 2 mA, 20 min daily	tPA/BDNF pathway in the hippocampus	Significantly increased the concentrations of tPA, BDNF, TrkB, and BDNF mRNA in the hippocampus.	(79)
CUMS rats	EA	DU20/EX-HN3	2 Hz, 0.6 mA, 30 min	/	Increased expression of BDNF /TrkB protein	(62)
CUMS rats	EA	GV20/PC6/SP6	Once daily for 14 d	/	Significantly increased BDNF expression in serum and hippocampus	(80)
High-intraocular pressure rats	EA	BL1/GV26	4/20 Hz, 1–2 mA, 15 min	DOR-BDNF/TrkB pathway	Increased the mRNA expression of DOR and TrkB Activate the DOR-BDNF/TrkB pathway	(86)
Insomnia model	EA	DU20/HT7	2/15 Hz,1 mA,30 min	cAMP/CREB/BDNF, PI3K/Akt pathways	Increased expression of BDNF	(87)

## 6. Mechanism of BDNF regulation by acupuncture

Acupuncture or EA is a type of stimulation; whether it stimulates the nerve’s main trunk or stimulates the nerve’s endings, it will cause the nerve to generate action potentials. The synthesis and release of neurotrophic factors and the transmission of information from receptors are regulated by action potentials. Therefore, acupuncture induces the central nervous system, especially the brain, to regulate the expression of neurotrophic factors, including BDNF. Acupuncture-induced neurotrophic factors trigger autocrine or paracrine signals that stimulate the production of adult neurons and can promote the activation of repair mechanisms and neural regeneration, resulting in a therapeutic effect on dysfunctions in neurological disorders (88).

From the multiple examples of acupuncture regulating BDNF listed in Section 5, acupuncture mainly affects the BDNF/TrkB signaling pathway by controlling the level of BDNF (as shown in Figure 4). Activating the downstream PI3K/Akt and MEK/ERK pathways affects neuronal growth and survival to promote nerve repair. Regulates plasticity by activating the PLC-γ pathway affecting CREB and thus LTP.

In addition, acupuncture promotes endogenous neurogenesis by mediating the upregulation of BDNF and the activation of related signaling pathways and by enhancing the proliferation, survival, and neuronal maturation of innate neural stem cells. Studies have revealed that neurogenesis takes place in the subventricular zone (SVZ) of the lateral ventricles in the adult brain (89), as well as in the sub granular zone (SGZ) of the dentate gyrus (DG) in the hippocampus (90). The SVZ is an essential source of endogenous cells, and neuroblasts migrate from the SVZ to the lesion site and differentiate into mature neurons that form new synapses (91). To build new neuronal circuits, these fully developed SGZ neurons move to the granule cell layer (90). Nevertheless, these increased neural progenitor cells /neural stem cells are not sufficient to sustain neurological recovery during ischemic conditions. Acupuncture-mediated BDNF promotion of adult neurogenesis in the brain may be an endogenous repair mechanism (92).

Acupuncture modulates the plasticity of synaptic structure and function. Acupuncture also affects BDNF, a protein associated with presynaptic and presynaptic structural plasticity. It has been found that EA improves synaptic plasticity and excitatory synaptic transmission efficiency, thereby improving learning and memory capacity by restoring LTP (93). Neuroplasticity may be a potential bridge between acupuncture and treating various nervous system diseases (94).

Moreover, the effect of acupuncture on glial cells (e.g., astrocytes and microglia) cannot be neglected. Astrocytes and microglia are glial cells, and the neurotrophic factors they release, including BDNF and NGF, is crucial for sustaining and fostering acupuncture-regulated neuroplasticity (67). From changes in synaptic coverage to the production of chemokines and cytokines to the release of dedicated “glio” transmitters, glial cells control synaptic plasticity in various ways. Glial cells can influence synaptic scaling, homeostatic plasticity, synaptic replasticity, meta-plasticity, LTP, and LTD (95).

Notably, we can find that acupuncture’s modulation of BDNF levels in other neurological diseases is upregulated, and only for neuropathic pain is BDNF levels downregulated. This is because the action of acupuncture on the BDNF pathway has a dual regulatory function of activation or inhibition. In neuropathic pain disorders, BDNF is involved in the initial sensitization of the pain pathway, and its increase early in the injury may be adaptive. However, sustained release of BDNF leading to excessive levels triggers maladaptive plasticity and harmful effects that can lead to neuropathic pain (96). Thus, acupuncture inhibits neuropathic pain by suppressing BDNF levels. In this review, BDNF expression is reduced in other neurological disorders, but acupuncture promotes neurological recovery by upregulating BDNF levels.

BDNF promotes neuroplasticity in the somatosensory cortex and facilitates hypersensitivity to neuropathic pain. By mediating LTP and controlling synaptic plasticity in the spinal cord, BDNF promotes central sensitization of the spinal cord. And central sensitization can cause neuropathic pain (88). Therefore, neuropathic pain is also reduced by inhibiting the central sensitization induced by the BDNF/TrkB signaling pathway.

At the same time, neuropathic pain is associated with microglial activation and elevated levels of BDNF, which induces activation of PI3K and ERK kinases in spinal microglia, and PI3K generates second messengers that activate phosphorylated-ERK. In microglia, ERK is activated after nerve injury and activated ERK underlies the development of neuropathic pain (97). Acupuncture inhibits ERK, a signaling pathway and key molecule activated by microglia during pain processing. EA attenuates hyperalgesia and allodynia associated with neuropathic pain by reducing microglia activation and BDNF expression (98). In addition, EA can interfere with pain transformation and is an effective treatment to prevent acute pain from turning into chronic pain.

## 7. Conclusion

In summary, this review found that acupuncture treatment modulates BDNF expression at multiple levels and participates in synaptic structural remodeling through the BDNF/TrkB signaling pathway, affecting neurogenesis and synaptic regeneration to rebuild damaged neural circuits. The action of acupuncture on the BDNF pathway has a dual regulatory function of activation or inhibition. In neuropathic pain, its activity is inhibitory; in other diseases, it is activating.

However, we mainly focused on the effect of acupuncture on the BDNF/TrkB signaling pathway, and the pro-BDNF/p75NTR signaling pathway was less studied, which can be explored in the future. Several neurological disorders reviewed in this paper are representative and widely studied. However, the regulation of BDNF by acupuncture is not limited to these diseases, and scholars will explore the effects of acupuncture on BDNF in more neurological disorders in the future. In addition, most of the studies in this review are basic studies, and relevant clinical application studies should be actively conducted to provide new therapeutic targets for the clinic by digging deeper into the intrinsic mechanism of acupuncture and moxibustion.

## Author contributions

CM designed an overview and wrote the manuscript. XL and YZ provided help and advice on writing, and modified the manuscript. All authors contributed to the article and approved the submitted version.

## Funding

This research was funded by the Heilongjiang Province “Leading Wild Goose” Team Project, grant number 2019-2.

## Conflict of interest

The authors declare that the research was conducted in the absence of any commercial or financial relationships that could be construed as a potential conflict of interest.

## Publisher’s note

All claims expressed in this article are solely those of the authors and do not necessarily represent those of their affiliated organizations, or those of the publisher, the editors and the reviewers. Any product that may be evaluated in this article, or claim that may be made by its manufacturer, is not guaranteed or endorsed by the publisher.

## Figures and Tables

**Table:** 

Akt (PKB)	protein kinase B (PKB) is also known as Akt
BDNF	brain-derived neurotrophic factor
CCI	chronic constrictive injury
CNS	central nervous system
CREB	cAMP-response element binding protein
CUMS	chronic unpredictable mild stress
DG	dentate gyrus
DRG	dorsal root ganglion
EA	electroacupuncture
ERK	extracellular signal–regulated kinases
GABAAR	γ-aminobutyric acid receptor type A
GDNF	glial cell line-derived neurotrophic factor
5-HT	5-hydroxytryptamine
HPA	hypothalamus-pituitary adrenocortical
JNK	c-Jun amino-terminal kinase
LTD	long-term depression
LTP	long-term potentiation
MAPK	mitogen-activated protein kinase
m-BDNF	mature brain-derived neurotrophic factor
MDD	major depressive disorder
MEK	mitogen activated protein kinase kinase
mGluR5	metabotropic glutamate receptor 5
MPTP	1-methyl-4phenyl-1,2,3,6-tetrahydropyridine
NAc	nucleus accumbens
NF-κB	nuclear factor kappa B
NGF	nerve growth factor
NMDA	N-methyl-D-aspartic acid receptor
PI3K	phosphoinositide 3-kinases
PKCε	protein kinase C epsilon
PLC-γ	phospholipase C-γ
pro-BDNF	proneurotrophin isoform of BDNF
p75NTR	p75 neurotrophin receptor
RhoA	Ras homolog gene family member A
RIRI	retinal ischemia–reperfusion injury
SGZ	sub granular zone
SH2	src homologous structural domain 2
SNI	spared nerve injury
SVZ	subventricular zone
tPA	tissue plasminogen activator
TrkB	tropomyosin-related kinase B
VTA	ventral tegmental area
